# Molecular detection of giant snakeheads, *Channa micropeltes* (Cuvier, 1831), one of the most troublesome fish species

**DOI:** 10.1038/s41598-021-89320-2

**Published:** 2021-05-11

**Authors:** Maslin Osathanunkul, Toshifumi Minamoto

**Affiliations:** 1 Department of Biology, Faculty of Science, Chiang Mai University, Chiang Mai, 50200 Thailand; 2 Research Center in Bioresources for Agriculture, Industry and Medicine, Chiang Mai University, Chiang Mai, Thailand; 3 Graduate School of Human Development and Environment, Kobe University, Hyogo, Japan

**Keywords:** Environmental impact, Biodiversity

## Abstract

A lack of reliable tools for determining the presence and distribution of fish species can impede understanding of predator–prey interactions and fishery management. Conventional fish survey methods are invasive, and can be size or species selective. Combining netting and electrofishing is a current method used to monitor fish species in Phayao Lake (Kwan Phayao), Thailand. However, the methods are inefficient and time-consuming. Recently, locals who rely on inland fisheries in Kwan Phayao expressed their deep concerns about the giant snakehead, *Channa micropeltes* (Cuvier, 1831) destroying other fish there. The giant snakehead prey on many commercially important fish species, as the prey species is reduced, negative effects on both biodiversity and the fishery sector could follow. Here, an eDNA-based survey was developed to detect the presence of the giant snakehead. Water samples were collected from six sites within Kwan Phayao and 17 sites in Ing River where water flowed into and out of Kwan Payao. The eDNA of the giant snakehead was detected in water samples from all collection sites using the developed qPCR assay with various concentrations. The eDNA was shown here to be a sensitive and reliable tool for fish surveillance so there will be a better chance for developing an effective management strategy.

## Introduction

In Thailand, inland capture fisheries are carried out principally in rivers, lakes, swamps and reservoirs. Phayao Lake (Kwan Phayao) is one of the major lakes used for inland fisheries^1^ and is reputed to be the 4th biggest lake of the country. The lake is home to about 50 fish species, many small-scale fishermen, and around 18,000 people from the city of Phayao. Many small-scale fishermen are self-employed and usually catch fish for direct consumption within their households or communities. Thus, small-scale fisheries tend to be firmly rooted in local communities, traditions and values and are very crucial in providing the rural poor with high quality protein food for home consumption.

Recently, there is an urgent need for a fast, reliable and sensitive approach for fish monitoring as it is not an invasive species that has caused huge problems but a troublesome, giant snakehead, *Channa micropeltes* (Cuvier, 1831). Because this species is native to Thailand, they have not gotten much attention until now. Locals who rely on inland fisheries in Kwan Phayao expressed their deep concerns about the giant snakehead destroying other fish there. The giant snakehead is not really capable of living in harmony with many other species as they can eat fish almost as big as they are. In Kwan Phayao, it is reported that they prey on many fish species such as Nile tilapia and Silver barb which are commercially important^2^. As the fish species which are prey of the giant snakehead are reduced, negative effects on both biodiversity and the fishery sector could follow.

A lack of reliable tools for determining the presence, distribution and abundance of fish species can impede understanding of predator–prey interactions and management of fish stocks^3^. Fish surveys in lakes can be a challenge because no single gear is capable of effectively sampling the wide range of habitats, each gear has specific selectivity with respect to size and species of fish (e.g.^4–8^). As a result, multiple methods are combined to complement each other for surveying fish communities^6^. Conventional fish survey methods are invasive and then can be harmful to the ecosystem^9^. The combining of netting and electrofishing is a current method used to monitor fish species in Kwan Phayao^2^. A survey of fish density and diversity by the Inland Fisheries Research and Development Division in 2009 found that electrofishing was ineffective for capturing partipentazona barb, *Puntius partipentazona* (Fowler, 1934) and spiny eel, *Macrognathus taeniagaster* (Fowler 1935), whereas netting was ineffective in sampling rohu, *Labeo rohita* (Hamilton, 1822), Asian redtail catfish, *Hemibagrus nemurus* (Valenciennes, 1840) and Kissing gouramis, *Helostoma temminckii* (Cuvier, 1829)^2^. These strongly indicated that the methods are size and species selective. In addition, in the past 3 years of fish surveys in Kwan Phayao (2016–2018), the giant snakehead was only detected in 2017 and no report of them was found during surveys in 2016 and 2018, although locals reported their present in the lake in both years^10^.

Therefore, the main limitations of the methods are their varying efficiency, incomplete or bias samplings, not to mention the fact that it is time-consuming. Recently, the use of environmental DNA (eDNA), defined as short DNA fragments that an organism leaves behind in environments, was proved to be a powerful tool in biodiversity science and conservation action which can monitor the biodiversity of a region in near real-time (e.g.^11–13^). Detection of the giant snakehead in Phayao Lake via eDNA instead of the traditional direct observation method could be more sensitive and less time consuming. In addition, eDNA monitoring of the giant snakehead could benefit the fish stocks management plan of Kwan Phayao as well as saving the small-scale fisheries there as strategies to obtain fishery metrics must appropriately and effectively acquire information needed to manage a fishery^14^.

## Results

### Species-specific primer design

Here, the primers were designed to amplify short target regions of 127 bp specific to the giant snakeheads (*C. micropeltes*). To determine specificity, the designed primers were tested in silico. The specificity of the generated primers was checked against the NCBI database by comparing the similarity of primers to *Channa* and related species. The homology of *Channa* sequences to the specific forward and reverse primers of *C. micropeltes* is 67.3–75.5% with at least five mismatches on the forward and four mismatches on the reverse primers (Table 1). When comparing with other fish species commonly found in Kwan Phayao, the homology was found to be lower (49.0–61.2%).

**Table 1:** Homology of the query to the forward and reverse primers, percentage identity as a function of the number of matching base sites divided by 47 (total number of base sites across the primer pair) Base site homology between the query and the primer is shown as a dot.

Species	Forward	Reverse	Identity, %	GenBank
*Channa micropeltes*	●●●●●●●●●●●●●●●●●●●●●●	●●●●●●●●●●●●●●●●●●●●●●●●●	100.0	KX129904
*Channa argus*	●●ACTTT●T●●●●●A●●G●●T●	●●●●●●CCC●●●●●●●●●●●●●C●–	71.4	GU937112
*Channa asiatica*	●A●CACATT●●A●●●●●G●●T●	●●●●●●TCC●●●●●●●●A●●●●C●–	67.3	KJ930190
*Channa gachua*	●●TC●C●TT●●C●●●●●G●●TT	●●●●●●TTT●●●T●●●●●●●●●C●–	69.4	NC 036,948
*Channa lucius*	–C●●●●●●T●T●●●●●●A●●T●	●●●●●●TAC●●●●●●●●A●●●●C●–	75.5	MF804538
*Channa maculata*	●●ACTTT●T●●●●●A●●G●●T●	●●●●●●CCC●●●●●●●●●●●●●C●–	71.4	KC823606
*Channa marulius*	●●TC●●●●T●●●●●●●●G●●T●	●●●●●●CC●●●●●●●●●●●●●●C●–	81.6	AB968638
*Channa striata*	–CTTATA●T●●●●●●●●G●●T●	●●●●●●CCC●●●●●●●●T●●●●C●–	67.3	KX177965
*Anabas testudineus*	●CAAACA●T●T●●●●●CA●CT●	●●●A●●T–AA●●●●●●●●●●●●C●G	61.2	NC 024,752
*Betta pi*	ACTAATTTT●●C●A●●CGCC●●	●●●●●●CCC●●●T●●●●●●●●●●T–	57.1	AB920288
*Betta splendens*	ACTAATTTT●TC●A●●CAC●●●	●●●●●●CCC●●●T●TG●A●C●T●●T	49.0	NC 026,581
*Chitala ornata*	ACTAATAAT●T●●A●●CCCC●T	T●●●●●C●C●●●–●TG●●●●AT●A–	49.0	NC 012,712
*Helostoma temminkii*	CCTAGCTTT●●C●●●●CGCCT●	●●●A●●C–CG●●●●●●●T●●●●●●–	55.1	NC 022,728
*Osphronemus goramy*	CCTAGCTTT●●C●●●●CACCT●	●●●●●T●CC●●●●●●●●●●●●●C●G	59.2	NC 031,363
*Trichogaster leerii*	TCTTA●TTT●●C●A●●CGCC●●	●●●●●●CT●●●●●●●●●T●●●●T●G	61.2	NC 026,725
*Trichopodus trichopterus*	TCTTTATAT●TC●A●●CGCC●●	●●●●●●TCC●●●●●●●●T●●●●C●G	55.1	NC 026,580

The qPCR assay with species-specific primers and probe showed that only DNA from the giant snakehead could be amplified, not other closely related fish species or species that are commonly found in Thai freshwater environments including Clown featherback, *Chitala ornata* (Gray, 1831), Great snakehead, *Channa marulius* (Hamilton, 1822), Jullien’s golden carp, *Probarbus jullieni* (Sauvage, 1880), Red tailed tinfoil, *Barbonymus altus* (Günther, 1868), Smith's barb, *Puntioplites proctozysron* (Bleeker, 1865) and Striped snakehead, *Channa striata* (Bloch, 1793).

### qPCR assay

The assay designed in this study was found to be species-specific to giant snakehead. Both PCR and qPCR did not result in any positive detection of the non-target species. The LOD and the LOQ of the assay determined by an analysis of the standard curve (Slope =  − 3.016, Y inter = 33.674, R^2^ = 0.976) generated from a dilution experiment under laboratory conditions. The LOD was 0.93 copies per reaction and the LOQ was 1.0 copies per reaction.

### Aquarium and field collection samples

An aquarium experiment was conducted to test the extent to which the qPCR of water samples can detect the giant snakehead at low simulated densities. The eDNA of the giant snakehead were detected from water samples collected at time 0, 3, 6, 12, 24, 48, 72, 96, 120, 144, and 168 h. after removing the fishes. Thus, the eDNA qPCR assay resulted in positive DNA signals in 100% of the aquarium experiment even 7 days after removal of the fishes from the tanks.

**Figure 1:**
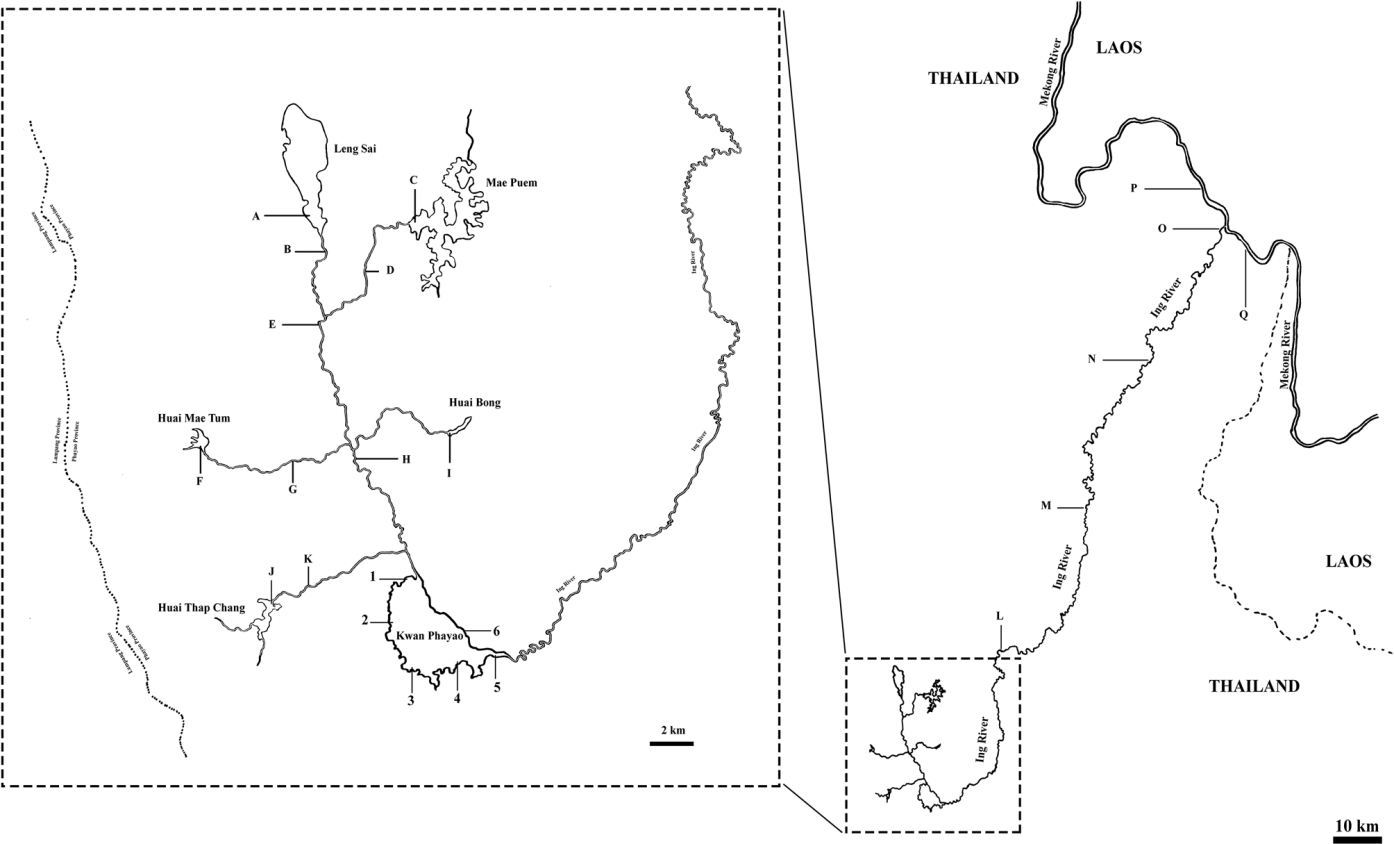
Map showing sampling sites. 1–6 water collecting sites in Kwan Phayao, A–K where water flowed into Kwan Phayao and L–Q where water flowed out of Kwan Phayao.

**Figure 2:**
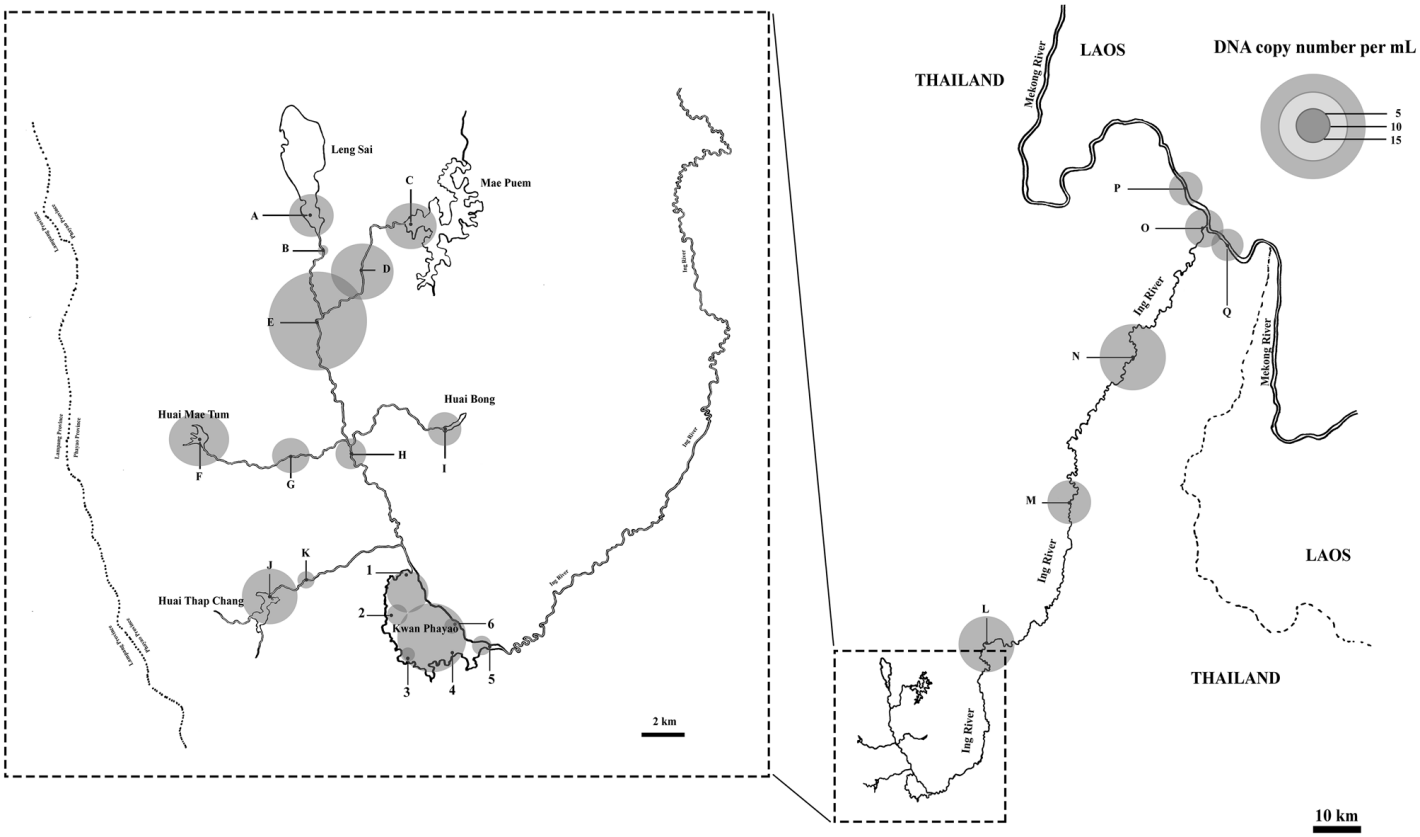
Map showing sampling sites with estimated concentration eDNA of the giant snakehead obtained from species-specific qPCR assay.

Water samples were collected from six sites at the same locations where the teams from the Inland Fisheries Research and Development Division, Department of Fisheries conducted the traditional surveys (Fig. 1). The eDNA of the giant snakehead were detected in water samples from all collection sites in Kwan Phayao using the qPCR assay (Fig. 2 and Table 2). There were no amplification detected in the negative controls. Concentration of the giant snakehead found in Kwan Phayao ranges from around 2.0–9.8 copy numbers per mL (Table 2).

**Table 2:** Average copy number and Ct (cycle threshold) obtaining form qPCR amplification of DNA samples from all sampling sites and standard fragments.

Site ID	Average copy number per mL	Average Ct
Positive tank	25.6	27.33
1	5.9	29.24
2	2.9	30.17
3	2.0	30.66
4	9.8	28.58
5	2.8	30.24
6	2.2	30.54
A	6.4	29.15
B	1.5	32.53
C	7.3	28.97
D	8.9	28.71
E	14.0	28.12
F	8.6	28.76
G	5.3	29.38
H	4.4	29.64
I	4.8	29.52
J	7.9	28.86
K	2.4	30.40
L	8.0	28.85
M	6.2	29.18
N	9.4	28.64
O	5.5	29.34
P	4.8	29.52
Q	4.6	29.57

Average values were calculated from three qPCR replicates with each replicate contain three replicates of each samples.

Further investigated Ing River which flows into and out Kwan Phayao was carried out. Water samples were collected from total of 17 sites (11 sites where water was going into Kwan Phayao and 6 sites where water was going out from Kwan Phayao to Mekong River). It is expected to detect the giant snakehead throughout the Ing River as there are no barriers stopping them from commodity. Also, the giant snakehead is now distributed all over Kwan Phayao with a large number that have an effect on other fish species. From qPCR assay with the water samples from Ing River, samples from all 17 sites were found to be positive in detecting the giant snakehead with various concentrations (Fig. 2). Concentration of the giant snakehead found in Ing River ranges from around 1.5–14 copy numbers per mL (Table 2). The highest concentration of the giant snakehead DNA outside Kwan Phayao was found at sampling site E (14 copy numbers per mL) which is higher than the highest concentration found in Kwan Phayao (site 4; 9.8 copy numbers per mL).

## Discussion

Recently, the use of environmental DNA (eDNA) has proved to be a powerful tool in biodiversity science and conservation action which can monitor the biodiversity of a region in near real-time. eDNA monitoring is likely to have a major impact on the ability of species detection. The ability to rapidly and sensitively detect the presence of a target species through eDNA analysis has enabled a wide range of scientific discoveries and technical advancements. One key of the success in eDNA detection of target species using qPCR is the specificity of primers and probe. In this study, the efficiency of specific primers and probe was tested. The primers and probe were confirmed to amplify only DNA from the giant snakehead, not other closely related fish species or species that are commonly found in Thai freshwater environments. As eDNA is thought to be easily degraded and presents itself in a short fragment, PCR primer sets used for eDNA studies should then be designed to yield short amplicons (100–180 bp)^15^. Also, it is noteworthy that short-amplicon PCR assays have high detection sensitivity^16^.

Several biotic and abiotic factors can affect the ability of eDNA detection such as breeding, feeding, seasonality, temperature, pH and potential natural inhibitors (e.g. algae, humic substances, and suspended sediment particles)^17–21^. To guarantee the success of eDNA qPCR experimenting, there are several ways to test for inhibitory effects such as using qPCR amplification of Internal Positive Controls^19^, using control DNA that known not be found in the sample^13^ and using DNA extraction kits with inhibitor removal steps^20^. Although there was report of algae found in Kwan Phayao^22^ which could act as inhibitors for our eDNA assays, we found no problem with the PCR amplification process here.

Previous traditional surveys of fish in Kwan Phayao failed to recognize the presence of the giant snakehead in some years and sampling sites (within Kwan Phayao) even when there were reports of locals finding and catching them from the lake. However, our method can detect the eDNA of the giant snakehead at all sampling sites in Kwan Phayao. Other works also proved eDNA detection is more sensitive than the conventional survey or sampling and, in many cases, it can be used for habitat selection and migration pattern study, biomonitoring the fish community and exploring genetic diversity^23–25^.

In addition, there were results from further investigation carried on at Ing River, which flows into and out Kwan Phayao. All sites were positive in detection of the giant snakehead DNA with various concentration. It indicated the expanding distribution of the fish. Even though Phayao authorities have several campaigns to reduce the number of the giant snakehead in Kwan Phayao, such as fishing competitions which reward fishermen a big prize to catch the most giant snakeheads in Kwan Phayao, and setting up training courses to educate locals in fish processing from the giant snakehead. Early detection monitoring of harmful species such the giant snakehead and others should be ranked among the top priorities in fisheries and conservation management due to the effects these organisms pose to ecosystems. The known ecological impacts of the giant snakehead include losing endangered species, altered structure and composition of aquatic communities, and a reduction in overall diversity of species. While long-term changes associated with the giant snakehead are being monitored through traditional surveys, it is also critical to be able to detect populations early. To the best of our knowledge, this is the first report of eDNA detection the giant snakehead in Thailand. The earlier they are found, the better the chances are of developing an effective management strategy. Early detection, rapid assessment and rapid response are critical defences against the establishment of population.

## Methods

### Ethics statement

All procedures were conducted in accordance with the current laws in Thailand on experimental animals and were approved by the safety management committee for experiments of the Laboratory Animal Center, Chiang Mai University (Project Number 2561/FA-0001). The study also followed the recommendations in the ARRIVE guidelines.

### Species-specific primer design

All the DNA tissue analysed originated from the mucus of the individual giant snakehead. Total DNA was extracted from the mucus sample using the Qiagen DNeasy Blood and Tissue Kit (Qiagen, Valencia, CA). Extracted DNA was used as a template for qPCR assay together with synthetic fragments. DNA samples were quantified using a Qubit fluorometer (Life Technologies) calibrated with the Quant-iT dsDNA HS Assay following the manufacturer’s instructions. For each replicate, 3 µL volumes were measured.

Species-specific primers and a minor-groove binding (MGB) probe incorporating a 5′ FAM reporter dye and a 3′ non-fluorescent quencher were designed to amplify an 127 bp targeting within the 16S region for the giant snakehead (*C. micropeltes*), using Primer Express (V3.0, Life Technologies; Table 3). Probe and primer sequences were matched against the National Centre for Biotechnology Information (NCBI, http://www.ncbi.nlm.nih.gov/) nucleotide database with BLASTn (Basic Local Alignment Search Tool) to confirm the species’ specificity for the giant snakehead in silico assays.

**Table 3:** Details of species-specific primers and the probe designed to amplify a 127 bp fragment of the 16S region of *Channa micropeltes* (Cuvier, 1831).

Primer name	Type	Length (bp)	Primer sequence 5′–3′
*Cmi-F*	Forward primer	24	CTCGCACCAACTAGGCTTTTCC
*Cmi-R*	Reverse primer	25	GGTTAATGTTCGGTGGATTGTCCGT
*Cmi*-*PR*	Probe	20	TGCTAACATGGAAGCACTTA

To ensure that the assay only amplified the giant snakehead, it was deployed on a closely related species commonly found in Thai freshwater environments using conventional PCR amplification and visualization on a 1.5% agarose gel stained with SYBR Safe DNA Gel Stain (Life Technologies).

### qPCR assay

The qPCR assay was deployed using Environmental Master Mix (Applied Biosystems) on mucus samples from the giant snakehead and related species to ensure the species specificity to the qPCR assay. In addition, eDNA qPCR assay for the giant snakehead, a water sample collected from tank at Phayao Freshwater Aquarium (Phayao Inland Fisheries Research and Development Center) was known to have only the giant snakehead was included as a positive control for the presence of amplifiable eDNA in water samples. The tank contains around 4.5 m^3^ of water with one individual of giant snakehead resides in the tank (the fish is about 60–70 cm in length).

All eDNA qPCR amplifications were performed in three replicates in a final volume of 20 µL, using 10.0 µL of 2 × TaqMan Environmental Master Mix 2.0 (Thermo Fisher Scientific), 2.0 µL of DNA template, 900 nM each of the F/R primers, and 125 nM of the probe. Samples were run under the following conditions: an initial 10 min incubation at 95 °C followed by 50 cycles of denaturation at 95 °C for 15 s and annealing/extension at 60 °C for 1 min. Negative controls with all PCR reagents but no template (three replicates) were run in parallel to assess potential contamination. The quantification cycle (Cq) was converted to quantities per unit volume using the linear regression obtained from the synthesized target gene standard curve (Integrated DNA Technologies Pte. Ltd., Singapore). The giant snakehead eDNA concentrations were then reported as copies/mL. The limit of detection (LOD) and the limit of quantification (LOQ) were also measured using the standard dilution series of synthesized target gene fragment with known copy numbers. A dilution series containing 1.5 × 10^1^ to 1.5 × 10^4^ copies per PCR tube were prepared and used as quantification standards. The calculation of LOD and LOQ was done using published R script by Klymus et al.^26^.

### DNA extraction from the filters

DNA trapped on the filters obtained from the aquarium experiments and field collections were extracted using Qiagen DNeasy Blood and Tissue Kit (Qiagen, Hilden, Germany) using a protocol modified from the manufacturer’s protocol with the following changes: the DNA from all samples were eluted twice with 25 µL AE buffer, in a total volume of 50 µL to obtain a more concentrated eDNA solution. The volume of ATL buffer (360 µL), Proteinase K (40 µL), AL buffer (400 µL) and Ethanol (400 µL) were doubled.

### Aquarium experiment

An aquarium experiment was used to test the extent to which qPCR of water samples can detect eDNA of giant snakehead at low simulated densities. The juvenile giant snakehead was obtained from the fish store and transported to a laboratory at Chiang Mai University. The giant snakeheads were then held in separate 120 L plastic holding containers in which the water was continuously filtered. The fish were fed frozen shrimp/commercially available flake fish food three times a week, and were held at 23 ± 1 °C.

The sensitivity of eDNA detection in the aquaria was evaluated by conducting three aquarium experiments using plastic tanks (30 × 45 × 25 cm) filled with 120 L of aged-tap water. The water in the tanks was continuously aerated through a filter. In each experiment, the giant snakeheads were randomly assigned to the tanks (10 individuals per tank). The average size of the snakeheads was 9.7 cm (body length ranging from 9.1 to 10.6 cm). The average weight was 8.15 g (ranging from 6.7 to 10.6 g). The water in the tanks was maintained at 23 ± 1 °C. A 300 mL water sample from each tank was collected at each time point (0, 3, 6, 12, 24, 48, 72, 96, 120, 144, and 168 after removal of the fishes from the tanks) in triplicate. Collected water was filtered on a GF/F filter (0.7 μm Whatman International Ltd., Maidstone, UK). The eDNA from each sample solution was extracted using a Qiagen DNeasy Blood and Tissue Kit (Qiagen, Hilden, Germany) in a final volume of 50 µL, detailed in *DNA extraction from the filters*. To confirm the absence of the giant snakehead eDNA in the water prior to the experiments, three tanks without giant snakehead were prepared and water sample was collected and treated as described above.

Real-time PCR was performed with the species-specific primers and probe set using a Rotor-Gene Q system (Qiagen, Hilden, Germany). The reaction conditions were the same as described in *qPCR assay*. Three replicates were conducted for each sample including the negative PCR control and positive control.

### eDNA field collection

Water samples were collected at 6 points within Kwan Payao according to the survey locations of the Inland Fisheries Research and Development Center. Additional water samples were collected from 11 and 6 locations in Ing River where water flowed into and out of Kwan Payao, respectively (Fig. 1). To avoid contamination, all field equipment was sterilized using 10% bleach, UV-Crosslinker or autoclaved and sealed prior to transport to the study site, and a separate pair of nitrile disposable gloves were used for each sample. At each site, water samples were collected three replicate in bucket that had been previously decontaminated with a 10% bleach rinse followed by two distilled water rinses.

In total, water samples were collected from 6 sites (in Kwan Phayao) and from 17 sites (in the Ing River) from 15th February to 5th March 2019, the middle of the dry season. Each site was sampled in triplicate, 300 mL samples of water were collected and filtered on GF/F filter (0.7 μm Whatman International Ltd., Maidstone, UK). In total, 306 water samples were collected from the surface water of lakes and rivers. For every sampling day, deionised water (300 mL) was filtrated as a negative control. The water samples and real-time PCR was processed as described above in *qPCR assay*.
